# The Charité protocol for surveillance, treatment and after-care management in women with Lynch syndrome

**DOI:** 10.1007/s00404-025-08112-5

**Published:** 2025-08-28

**Authors:** Lukas Chinczewski, Radoslav Chekerov, Severin Daum, Claus-Eric Ott, Jalid Sehouli

**Affiliations:** 1 Department of Gynecology With Centre of Oncologic Surgery, Charité – Campus Virchow-Klinikum, Charité – Universitätsmedizin Berlin, Augustenburger Platz 1, 13353 Berlin, Germany; 2 Department of Gastroenterology, Infectious Diseases and Rheumatology, Charité - Universitätsmedizin Berlin, Hindenburgdamm 40, 12200 Berlin, Germany; 3 Institute for Medical and Human Genetics, Campus Virchow-Klinikum, Charité - Universitätsmedizin Berlin, Augustenburger Platz 1, 13353 Berlin, Germany

**Keywords:** Lynch syndrome, Gynecolocigal cancer, surveillance, hereditary carcinoma, women's health

## Abstract

**Background:**

Lynch syndrome (LS) is the most common inherited cancer syndrome, caused by germline mutations in mismatch repair (MMR) genes such as MLH1, MSH2, MSH6, and PMS2. While primarily associated with colorectal cancer, LS significantly impacts gynecological oncology, with increased risks for endometrial and ovarian cancers. Despite its clinical relevance, structured counseling and surveillance programs tailored to LS patients in gynecology are lacking.

**Objective and methods:**

This study presents the first structured gynecological outpatient consultation program for LS patients in Germany, established at Charité—Universitätsmedizin Berlin in August 2021. The aim was to develop an individualized, multidisciplinary framework for surveillance, therapy, and follow-up care, addressing the specific needs of different patient cohorts. Between August 2021 and December 2023, clinical data from 40 LS patients were collected and analyzed descriptively. From this experience, we furthermore concluded a guideline for the care of individuals with Lynch syndrome.

**Results:**

Among the 40 patients, 21 had been diagnosed with cancer (affected group), while 19 were cancer-free and undergoing routine surveillance (non-affected group). The distribution of MMR gene mutations was 40% MSH2, 25% MSH6, 25% PMS2, and 15% MLH1. In the non-affected group, the median age was 38 years, with a BMI of 21.4. Surveillance identified one urothelial carcinoma and one case of endometrial hyperplasia. In the affected group, the mean age was 55.2 years, and the BMI was 24.7. Twenty-three gynecological cancers were diagnosed, of which 52% were endometrial, 26% ovarian, and 18% breast cancers. 61.1% of tumors were MSI-positive, and 33.3% of patients received immunotherapy.

**Conclusion:**

A holistic, multidisciplinary approach is essential for the management of LS patients in gynecological oncology. The structured consultation model developed at Charité facilitates personalized surveillance, risk-adapted prevention, and evidence-based therapy strategies. Future studies and clinical trials should further investigate screening protocols, therapeutic interventions, and the role of LS patients in targeted treatment approaches. This guideline serves as a preliminary framework and will be continuously adapted as new research emerges.

## What does this study add to the clinical work

**Table:** 

This article or study adds significant benefit to clinical work since it is a first holistic, interdisciplinary protocol for the care of patients or women with lynch syndrome. Not only surveillance but also therapy and after-care should follow a specialized protocol since this cohort has special and complex needs.	

## Introduction

Lynch syndrome, also known as hereditary non-polyposis colorectal cancer (HNPCC), is the most common inherited cancer syndrome and is caused by germline mutations in DNA mismatch repair (MMR) genes, such as MLH1, MSH2, MSH6, and PMS2. While Lynch syndrome is most frequently associated with colorectal cancer, its implications extend significantly into gynecology, particularly in the development of endometrial and ovarian cancers. Within the last decade and latest with the introduction of molecular subtypes in endometrial cancer, Lynch syndrome is focused on more and more in gynecological treatment [1–3].

Endometrial cancer is the most common extracolonic malignancy in individuals with Lynch syndrome, with approximately 2–5% of all endometrial cancers being attributed to this genetic condition [4, 5]. The prevalence of Lynch syndrome in the subgroup of MSI-high tumors is described with approximately 10% [6]. Similarly, around 1–3% of ovarian cancers are linked to Lynch syndrome, underscoring the critical need for awareness and tailored management strategies in gynecological practice [2, 7, 8]. These cancers often present at a younger age in Lynch syndrome patients compared to sporadic cases, further emphasizing the importance of early detection and preventive measures [6, 9].

Screening plays a pivotal role in the management of Lynch syndrome [10, 11]. Strategies such as regular transvaginal ultrasound and endometrial biopsy can aid in the early detection of gynecological malignancies in high-risk individuals and are recommended in ESGO guidelines. Furthermore, germline testing for MMR mutations in EC is mandatory in identifying at-risk patients and guiding appropriate surveillance. Prophylactic measures, including hysterectomy and bilateral salpingo-oophorectomy, may also be considered for mutation carriers to significantly reduce cancer risk.

Given its genetic basis and the availability of effective screening and preventive strategies, Lynch syndrome represents both a challenge and an opportunity in gynecological oncology. Early identification of carriers and consequent surveillance may substantially improve patient outcomes and prevent the development of cancer. Due to this multilayered screening, individuals need a structured and centered consultation.

Established in August 2021, our Lynch Syndrome Consultation Program aims to provide personalized and holistic care to individuals affected by or at risk for Lynch syndrome. It combines thorough patient education, targeted preventive measures, and evidence-based treatment planning to address the unique needs of this patient group. Below is an in-depth overview of the program’s objectives, patient cohort, consultation content, and follow-up procedures. Within this article, we would like to present our first experiences.

### The first gynecological outpatient counseling for individuals with Lynch syndrome in Germany

#### Program goals and patient cohort

In August 2021, the first Germany-wide outpatient clinic concentrating on Lynch syndrome was established at our gynecological department in Berlin. The primary goal of this consultation hour is to deliver a comprehensive framework for managing Lynch syndrome, including preventive care, early detection, and personalized therapeutic planning. For this differentiated approach, patients need to be put in the following different cohorts:

1. The healthy mutation carrier: preventive measures and surveillance
2. The cancer patient undergoing therapy: therapy optimization and genetic aspects
3. The cancer patient in follow-up care: risk management and recurrence prevention
4. The cancer patient without genetic testing: clinical indications and diagnostic strategies.

Through this differentiated approach, patient care should be individualized, holistic, and disease-specific to meet the high demands of prevention and follow-up care.

In this paper, we will present our first analysis of this counseling hour and conclude a differentiated approach for surveillance, therapy, and after-care approach.

## Material and methods

Clinical patient data were collected between August 2021 and December 2023 and systematically recorded in a table consecutively by occurring patients. The dataset was subsequently analyzed using descriptive statistical methods. Mean and mode values were calculated to summarize the data. This study is purely a descriptive analysis of the collected data. For the following therapy, surveillance, and aftercare recommendations, we conducted research and combined the findings with our own clinical experience to develop practical recommendations.

### First results: a descriptive analysis of findings

Between August 2021 and December 2023, data were collected from a total of 40 patients attending the clinic for Lynch syndrome screening and management. Among these, 21 patients had been diagnosed with cancer (affected group), while 19 patients remained cancer-free and attended regular surveillance appointments (non-affected group).

The distribution of Lynch syndrome-related germline mutations divides in the following: 40% in MSH2, 25% in MSH6, 25% in PMS2, and 15% in MLH1. Patients reported a median of 3 [0;5] affected family members with Lynch syndrome-associated cancers.

In the non-affected group, median age was 38 years [25;51] and the average BMI was noted as 21.4 [18.96; 23.82]. In terms of reproductive history, the average gravida count was 0.88, and the average para count was 0.73. On average, 0.47 medications were taken. Regarding reproductive health, the average menstrual duration among these patients was 27.9 days, with a mean cycle length of 5.53 days. The age of diagnosis of Lynch syndrome was, on average, 32. Regarding comorbidities, 4 patients showed thyroid dysfunction and 3 patients had diagnosed endometriosis. Patients attended a total of 29 surveillance examinations during the study period. 6 patients already had a tumor diagnosis, among these, 4 with colorectal cancer and 1 with gastric cancer. During our routine screens, one patient was diagnosed with urothelial carcinoma due to the routine transvaginal ultrasound. She did not have any symptoms. One patient showed endometrial hyperplasia.

Focusing on the affected group, mean age at presentation was 55.2. The BMI was 24.7 [19.9;29.5]. The number of patients who were diagnosed with Lynch syndrome because of a gynecological or a non-gynecological tumor was 9. Medication use varied, with a median of 2 medications taken, ranging from 0 to 11.

In total, there were 23 cases of gynecological cancer diagnosis in the cohort. Twelve of these were EC (52%), six of them OC (26%), four were breast cancer (18%) and one patient was diagnosed with cervical cancer (4%). Overall, 50.0% of the cases had a FIGO staging of FIGO I; meanwhile, FIGO II was seen in none of the cases. FIGO III was at 16.7%. There was no FIGO IV. In terms of grading, 22.2% of the 18 cases showed a grading of G1, 38.9% G2, and 33.3% G3, respectively. Regarding immunohistochemistry, 61.1% of the tumors tested positive for MSI, and only 5.56% of the tumors were negative. For the remaining 33.3% (*N* = 6), these data were not available. In terms of treatment choice, 94.5% underwent surgery, 61.1% received chemotherapy, and 22.2% radiotherapy. Immunotherapy was chosen in 33.3% of the patients. Differentiated data on EC and OC can be seen in Table 1.

**Table 1:** Patients Characteristics of the gynecological cancer affected and non-affected group

		Non-affected group (*N* = 19)	Affected group (*N* = 12)	
			Endometrial Cancer	Ovarian cancer
*N*		19	7	5
Age (mean)		38,6	53.6	46.6
BMI (mean)		21.34	25.3	24.7
Gene mutation	MSH2	8 (42%)	3 (43%)	2 (40%)
	MSH6	4 (21%)	3 (43%)	1 (20%)
	MLH1	3 (16%)	1 (14%)	0 (0%)
	PMS2	4 (21%)	0 (0%)	2 (40%)
Medications (mean N)		0.47	2.26	
Gravida / Para (mean)		1 / 0.83	1.36 / 0.83	
Cycle length/menstruational duration (mean d / d)		27,7 / 5,2	N/A	
Non-gynecological cancers (total N)		7	10	
Prophylactic HE + BSO performed		5 (26%)	N/A	
Stadium FIGO	I		5 (71.4%)	3 (60%)
	II		1 (14.3%)	0 (0%)
	III		1 (14.3%)	2 (40%)
	IV		0 (0%)	0 (0%)
Grading	G1		3 (43%)	0 (0%)
	G2		3 (43%)	1 (20%)
	G3		1 (14%)	4 (80%)
MMR-status	dMMR		5 (100%)	3 (100%)
	pMMR		0	0
Therapy	Surgery		7 (100%)	5 (100%)
	Syst. LNE		7 (100%)	5 (100%)
	Positive LN		0 (0%)	2 (40%)
	Chemotherapy		1 (14%)	5 (100%)
	Radiation		2 (28%)	0 (0%)
	Immunotherapy		2 (28%)	1 (20%)

Within this table Lynch-like tumors (MMRd without a germline mutation) are excluded *BMI* Body mass index, *dMMR* deficient mismatch-repair system, *pMMR* proficient *MMR*, OP surgery, *Syst.* *LNE* systematic lymphonodectomy, *LN* lymph nodes

In total, 19 comorbidities were found in this cohort: 34% of these were of endocrinological nature, followed by 13% psychosomatic/psychiatric diagnosis. 12% were cardiovascular diseases. 4 out of 19 patients (21.1%) in the cohort were found to have an additional non-gynecological tumor diagnosis. With two of the patients having more than one non-gynecological tumor diagnosis in their history, a total of an additional eight tumor cases were detected in the cohort, four being a GI-Tract tumor (e.g., colorectal carcinoma), two being a peritoneal carcinoma, one being a glioblastoma, and lastly, one being a basalioma (Table 2).

**Table 2:** Landscape of extra-gynecological cancer (in both cohorts)

Tumor site	Histology	Treatment	
Glioblastoma	N/A	OP + RTX + Temozolomid	PMS2-mutation
Synchronic jejunal, colon and rectal carcinoma	Jejunal: pT2m pN0 (0/88) G2 L0 V0 Pn0 R0 Colon: pT2 Rectal: pT1	OP	NRAS-wt
Jejunal cancer	pT3 pN1 (1/1) G2 L0 V0 Pn0 R0; UICC IIIA	OP + CTX	N/A
Colon ascendens with peritoneal metastasis	pT4b pN1a (1/18) pM1c (PER) G3 RX L0 V0 Pn0	OP + ITX + HIPEC	Loss of MLH1/PMS2, CPS 0, BRAF-wt, KRAS-wt, NRAS-wt
Colon	pT3 pN1b (2/28) G3 R0 L1 V1 cM0	OP + CTX	Loss of MSH2/MSH6
Basalioma	N/A	OP	N/A
Sigmoid	pT3 pN0 R0	OP	N/A
Appendix	pT4 pN0 cM0	OP + CTX	
Gastric	N/A	OP	N/A
Cecum	pT3 pN0 cM0 l0 Pn0 R0; UICC IIA	OP	N/A
Urothelial	pTa G2	OP	N/A
Rectal	cT3 N + M0	ITX	MSI

*OP* Operation; *CTX* Chemotherapy; *ITX* Immunotherapy; *RTX* Radiotherapy; *wt* Wild-type; *CPS* Combined positive score; *HIPEC* Hyperthermic intraperitoneal chemotherapy; *MSI* Microsatellite instability

### Charité guideline for the management of patients with Lynch syndrome

**Fig. 1:**
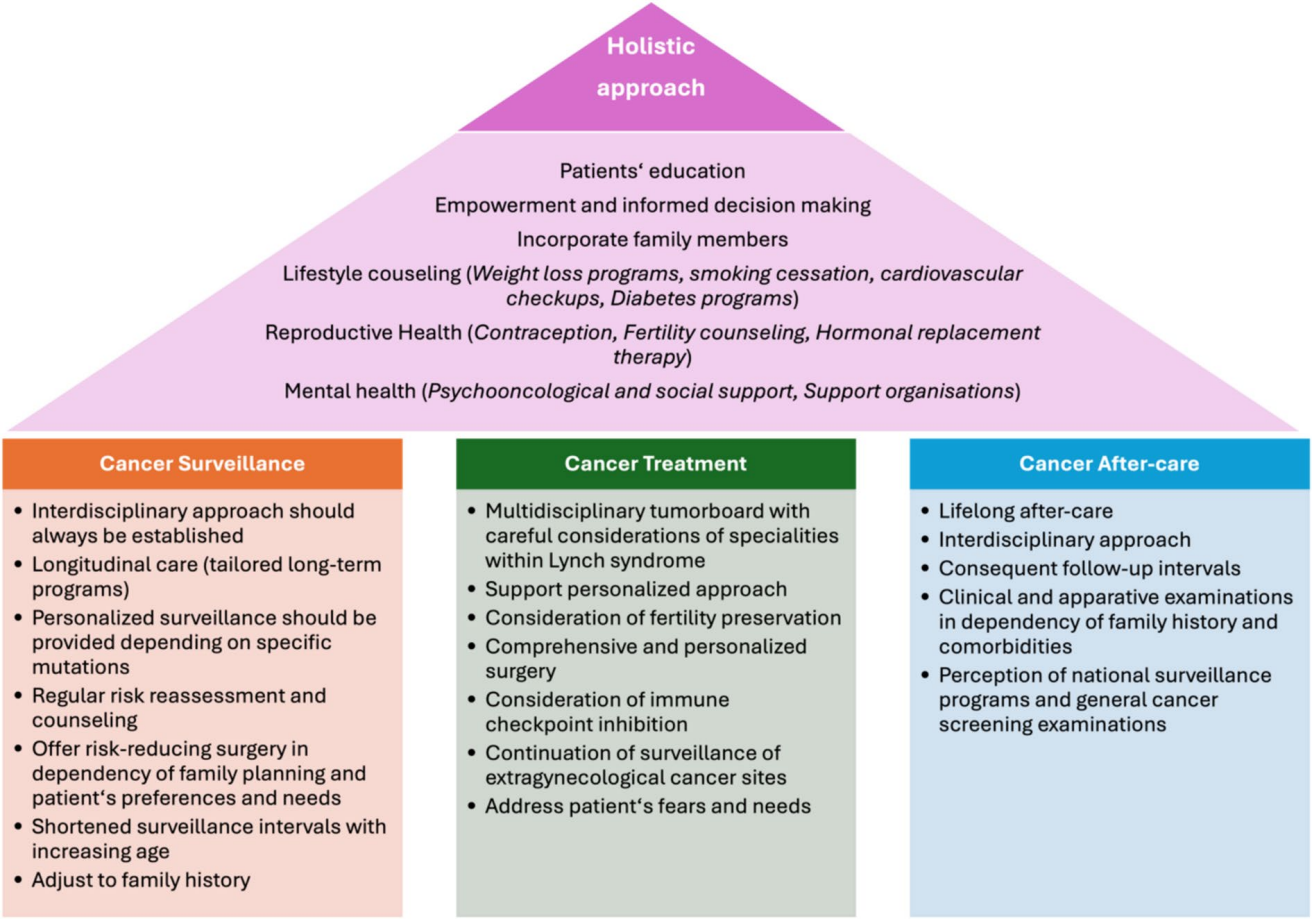
The House of Comprehensive Lynch Syndrome Care: a holistic framework covering surveillance, treatment, and after-care

Concluding from our first results and our clinical experiences, we developed a differentiated approach for the care of patients with Lynch syndrome, which is presented in the following. Therefore, we divided patients into different cohorts, as described above (Fig. 1).

#### Basic concept for all mutation carriers

Patients with Lynch syndrome represent a unique and complex cohort, posing a significant challenge to interdisciplinary medical care. As carriers of a hereditary cancer syndrome, they face lifelong uncertainty and heightened anxiety regarding their personal cancer risk and that of their families. Beyond medical surveillance and treatment, these patients require structured, proactive guidance from healthcare providers to navigate their diagnosis and long-term care. A coordinated and well-structured care concept is essential to establish trust and clarity, ensuring that patients feel supported in making informed decisions. By integrating multidisciplinary expertise, medical teams can provide a comprehensive and individualized roadmap, allowing patients to move forward with confidence and a sense of control over their health journey.

Our considerations are:

- The tumor predisposition syndrome should be explained to all patients in a comprehensive and differentiated manner, emphasizing its relevance for both the affected individual and their relatives.
- As part of a holistic approach, patients should be informed about general risk factors for cancer development. Lifestyle counseling should always be offered, with a strong emphasis on patient education. Patients should be given access to risk reduction programs, such as smoking cessation or weight loss programs. During consultation a detailed discussion about surveillance programs, including their pros and cons, guided by the latest scientific evidence is mandatory to empower the patients for an informed decision making on preventive examinations. We are also empowering patients to recognize symptoms or “red flags” for gynecological cancers, such as: irregular bleeding (e.g., menometrorrhagia, postmenopausal bleeding) or Dysmenorrhea, unexplained abdominal pain or swelling, unintended weight loss. Guidance on when to seek medical evaluation is explained.
- A detailed medical history is mandatory for an individual therapy approach. A comprehensive review of personal medical history is always done, including: previous illnesses, risk factors, menstrual history, endometriosis, and contraceptive use; family history with a focus on cancer and hereditary patterns; fertility concerns and future reproductive planning.
- Comorbidities should be continuously evaluated and adequately managed, with regular reassessments by the treating physicians. A connection to appropriate medical specialties should be ensured. Referral to additional medical specialties should be initiated and monitored as needed.
- Access to psycho-oncological and psychosocial support should be provided.
- Patients should be informed about relevant support organizations. In Germany patients advocacies such as SemiColon (https://www.semi-colon.de), the German Ovarian Cancer Foundation (https://stiftung-eierstockkrebs.de/willkommen/), and the Gyn-Oncology Forum (https://forum-gyn-onkologie.de) should be presented. Appropriate informational materials should be made available.
- Structured documentation of all performed examinations should be ensured.

#### The healthy mutation carrier

Patients with Lynch syndrome undergo intensified cancer surveillance, facing a lifetime of heightened medical attention due to their increased genetic predisposition. Despite the limited evidence base guiding optimal surveillance strategies, it remains crucial to acknowledge and address their cancer-related anxiety. A standardized, yet highly individualized approach is essential—balancing evidence-based recommendations with personalized risk assessment and patient preferences. Surveillance should not only aim for early cancer detection but also provide reassurance and empowerment, ensuring that patients feel actively involved in their long-term health management.

Our considerations are:

- All patients with a confirmed germline mutation in Lynch syndrome-associated genes should be offered an intensified surveillance program.
- A critical discussion regarding gynecological surveillance should take place with each patient before inclusion in the screening program.
- The exact approach should always be tailored to the specific germline mutation, taking into account the patient’s preferences, family history, and the most recent scientific literature.
- Since long-term follow-up is required, regular reassessments and risk counseling should be conducted at appropriate intervals.

#### The cancer patient undergoing therapy

Patients with Lynch syndrome-associated cancers represent a unique cohort in oncological care, requiring a precise identification of the tumor-driving mechanisms to tailor therapeutic strategies accordingly. Unlike sporadic malignancies, the molecular landscape of Lynch syndrome tumors often dictates distinct treatment considerations, particularly in the context of immune checkpoint inhibition and targeted surgical interventions. To ensure optimal management, multidisciplinary tumor boards play a crucial role. These boards should comprise experts who are well-versed in the specificities of Lynch syndrome, allowing for a nuanced evaluation of treatment options. Beyond standard oncological approaches, therapy should be highly personalized, integrating the holistic care model by addressing not only oncological, but also reproductive, psychosocial, and long-term surveillance aspects (Table 3).

**Table 3:** Recommendations for the Intensified surveillance program for Lynch and Lynch-like syndroms

		Lynch syndrome				Constitutional MMR deficiency^a^	Familiar colon cancer type X^b^
		MLH1	MSH2	MSH6	PMS2		
Median age onset [9]	CRC	56	56	61	66	N/A	N/A
	EC	52	52	60	61	N/A	N/A
	OC	49	47	66	58	N/A	N/A
Gastroenterological surveillance ^e)^							
	Abdominal sonography	annual starting at age 25				Individually	Like Lynch
	Endoscopy	every 1–3 years starting at age 25				Individually	Like Lynch
	Colonoscopy	every 1–2 years starting at age 25				Individually	Like Lynch
	Skin screening	every 2 years				Individually	Like Lynch
Gynecological surveillance							
Age Onset*		35	30	40	Not necessarily warranted	Teen age, if known	5 years before the age of diagnosis of the index patient
Interval		(bi-)annually			every 1–2 years	(bi-)annually	Every 1–2 years depending on family history
Content	Patients history	✓	✓	✓	✓	✓	✓
	Risk advice	✓	✓	✓	✓	✓	✓
	Clinical examination	✓	✓	✓	✓	✓	✓
	Speculum setting	✓	✓	✓	✓	✓	✓
	PAP test + HPV typing	In accordance with general precautionary recommendations					
	TVUS^g^	✓	✓	✓	✓	✓	✓
	Endometrial pipelle / Office hysteroscopy^f^	✓	✓	✓	(✓)	✓	Only if EC in family history
	CA-125 levels^e^	✓	✓	✓	(✓)	∅	∅
	Urine stix	(✓)	✓	(✓)	∅	✓	∅
	Renal sonography	(✓)	✓	(✓)	∅	✓	Only if renal cancer in family history
	Breast examination	✓	✓	✓	✓	✓	✓
	Mammography or sonography	∅	∅	∅	annually	Only within screening programs	Only within screening programs
Prophylactic HE + BSO^c^		Offer at age 40			Offer at age 45	Offer at age 40	∅
HRT^d^		✓	✓	✓	✓	✓	

*CRC* Colorectal cancer, *EC* Endometrial cancer, *OC* Ovarian cancer; *N/A* Not available; *TVUS* Transvaginal sonography; *HE* Hysterectomy; *BSO* Bilateral salpingo-oophorectomy; *HRT* Hormone replacement therapy

^*^in analogy to updated ESGO guideline for endometrial cancer 2025

^a^Autosomal recessively inherited form with early onset, including glioblastomas already occurring in childhood and adolescence

^b^Fulfilling clinical criteria without clear detection of a mutation—a critical reassessment of genetic testing every two years is recommended (e.g., reclassification of variants of uncertain significance)

^c^The prophylactic surgery must always be discussed carefully with the patient and must be performed in dependency of the patients’ wishes and needs. Before hysterectomy (HE), always perform hysteroscopy and biopsy to rule out an existing, asymptomatic carcinoma. Oophorectomy should always be critically discussed in younger patients. If ovarian preservation is chosen, the procedure should always include HE + bilateral salpingectomy (since ovarian cancer may rise from STIC cells). A two-step approach is possible. Uterine morcellation is strongly not recommended. Hormonal replacement therapy can always be conducted if no cancer was found in the pathological examination

^d^Only perform HRT up to a maximum age of 50 after BSO. Before BSO: recommendation for bone density measurement as a baseline assessment

^e^CA-125 levels are not recommended even in risk populations in cancer guidelines. The data were primarily collected in a cohort of BRCA mutation carriers. Therefore, we included CA-125 levels in our recommendation since the levels remain unclear in Lynch syndrome. It may be discussed individually with the patient

^f^(bi-)annual pipelle or hysteroscopy must be discussed individually with the patient. We usually conduct in a first examination the office hysteroscopy for a baseline examination. It may be reperformed in the following years. There is no clear data yet, whether hysteroscopy or pipelle performs better

^g^during TVUS it is important to also have a look at the urine bladder to exclude bladder cancer. There is no general recommendation for the conduction of a cystoscopy. If urine stix and/or the vaginal sonography show suspicious lesions, referral to urology should be conducted

Our recommendations are:

- Fertility preservation options should be evaluated for all patients before the start of therapy.
- Comprehensive surgical assessment of lymph nodes and other high-risk regions is essential to accurately determine disease spread.
- Patients with MMR deficiency or MSI-high status often respond well to immune checkpoint inhibitors. This is particularly relevant in palliative or refractory settings.
- In locally advanced or metastatic endometrial cancer, immune checkpoint inhibition should be considered as part of primary therapy.
- For all ovarian cancers, regardless of histological type, disease stage, or timing of treatment, the use of checkpoint inhibition should always be evaluated [3].
- Routine screening and preventive examinations in other medical specialties (e.g., gastroenterology) should continue during the treatment of gynecological cancer. Regular reassessment by treating physicians during therapy is essential. These screening should not be omitted even during checkpoint inhibition therapy.
- Hormone replacement therapy (HRT) should be provided to young patients. In this case, the primary driver of malignancy is Lynch syndrome, not hormone receptors.

#### The cancer patient without genetic testing

In clinical practice, it is essential to actively consider the possibility of an underlying tumor predisposition syndrome, particularly in patients diagnosed with Lynch syndrome-associated malignancies. Many patients are unaware that their cancer could be linked to a hereditary condition, making it our responsibility as healthcare providers to identify potential risk factors and initiate further evaluation when appropriate. A thorough family history assessment and tumor analysis should be standard practice, as they can provide crucial indicators for genetic predisposition. Early identification of Lynch syndrome not only impacts the patient’s treatment and surveillance strategy but also allows for preventive measures in at-risk family members. Recognizing and addressing hereditary cancer risks is a fundamental step toward personalized and proactive patient care.

General considerations:

- A detailed family history must be obtained for all cancer patients. The PREMM5 model from Harvard University (https://premm.dfci.harvard.edu) can be used for preliminary risk stratification to determine the indication for genetic testing.
- If genetic testing reveals a variant of uncertain significance (VUS) in Mismatch-Repair (MMR) genes, its potential pathogenicity should be reassessed at regular intervals.
- Patients with a VUS may be offered intensified surveillance, provided that the family history suggests a hereditary predisposition.
- If a pathogenic variant is identified, predictive testing should be offered to at-risk relatives.

Endometrial cancer:

- During the diagnostic workup of endometrial cancer, immunohistochemical (IHC) staining for MMR protein loss should already be performed on the curettage specimen.
- If IHC shows a loss of MSH2 and/or MSH6 or isolated PMS2, genetic testing is always indicated.
- If MLH1/PMS2 loss is detected, DNA promoter hypermethylation analysis should be performed to differentiate between sporadic and hereditary cases.
- A normal IHC result does not override a suspicious family history.

Ovarian cancer:

- In cases of endometrioid ovarian cancer, immunohistochemical (IHC) testing for MMR protein loss should be performed. The same approach as for endometrial cancer applies.
- Every patient with ovarian cancer should undergo at least BRCA genetic testing (as recommended by the consortium). It is essential to ensure that the panel used also includes Lynch syndrome-associated genes, which are typically part of standard BRCA panels.

#### The cancer patient in follow-up care

Patients in tumor aftercare present a unique challenge in the management of Lynch syndrome. While they may have overcome their initial cancer diagnosis, their lifelong predisposition to developing secondary malignancies remains a critical concern. Unlike patients with sporadic cancers, their journey does not end with remission—it requires ongoing vigilance, tailored surveillance, and proactive risk management. As specialists, it is our responsibility to look beyond our own field of expertise and ensure that patients receive comprehensive follow-up care that addresses their full spectrum of cancer risks. A multidisciplinary approach is essential to prevent fragmented care and ensure that surveillance, prevention, and psychosocial support remain integral components of their long-term health strategy.

Our considerations are:

- For gynecological malignancies, we recommend lifelong continuation of intensified follow-up care. It is crucial to ensure comprehensive cancer screening, which must be regularly monitored by the treating physicians.
- Follow-up intervals should adhere to standard guidelines.
- Follow-up examinations for gynecological patients (endometrial and ovarian cancer) should include the following:
  o Abdominal palpation and speculum examination
  o Transvaginal ultrasound, including assessment of the bladder wall and vaginal vault
  o Transabdominal ultrasound, including evaluation of the kidneys
  o Monitoring of the tumor marker CA-125
- Gynecological follow-up should not be discontinued after the prescribed 5-year period. Annual screening and follow-up examinations are recommended and can be structured individually with the patient.
- In cases of early-stage ovarian cancer with uterine preservation, tissue biopsy should continue via endometrial sampling (pipelle biopsy) or office hysteroscopy.

## Conclusion

The management of patients with Lynch syndrome requires a holistic therapeutic approach that integrates multidisciplinary collaboration across various medical specialties. Given the complexity of this tumor predisposition syndrome, a highly differentiated and individualized strategy is essential to ensure optimal prevention, therapy, and follow-up care.

Our Lynch Syndrome Consultation Program exemplifies a holistic and patient-centered approach to healthcare. By addressing not only the medical but also the emotional and social aspects of Lynch syndrome, it ensures comprehensive, individualized care. This approach aims to empower patients, optimize early detection, and tailor interventions, reflecting the program’s commitment to improving patient outcomes and quality of life. This guideline serves as a framework to structure clinical decision-making but must be continuously adapted as new insights emerge.

Ongoing research is needed to further evaluate and refine screening and surveillance protocols, ensuring that patients receive evidence-based and risk-adapted care. Additionally, Lynch syndrome cohorts should be more systematically included in clinical drug trials to assess tailored treatment strategies and potential benefits of novel therapeutic approaches.

## Data Availability

Data are provided within the manuscript.
